# Importance of Reciprocal Balance of T Cell Immunity in *Mycobacterium abscessus* Complex Lung Disease

**DOI:** 10.1371/journal.pone.0109941

**Published:** 2014-10-08

**Authors:** Su-Young Kim, Won-Jung Koh, Yee Hyung Kim, Byeong-Ho Jeong, Hye Yun Park, Kyeongman Jeon, Jong-Seok Kim, Sang-Nae Cho, Sung Jae Shin

**Affiliations:** 1 Division of Pulmonary and Critical Care Medicine, Department of Medicine, Samsung Medical Center, Sungkyunkwan University School of Medicine, Seoul, South Korea; 2 Department of Microbiology, Institute of Immunology and Immunological Diseases, Brain Korea 21 PLUS Project for Medical Science, Yonsei University College of Medicine, Seoul, South Korea; University of Minnesota, United States of America

## Abstract

**Background:**

Little is known about the nature of the host immune response to *Mycobacterium abscessus* complex (MABC) infection. The aim of the present study was to investigate whether alterations in serum immunomolecule levels after treating MABC lung disease patients with antibiotics can reflect the disease-associated characteristics.

**Methods:**

A total of 22 immunomolecules in 24 MABC lung disease patients before and after antibiotic therapy were quantitatively analyzed using a multiplex bead-based system.

**Results:**

In general, the pre-treatment levels of T helper type 1 (Th1)-related cytokines, i.e., interferon (IFN)-γ and interleukin (IL)-12, and Th2-related cytokines, i.e., IL-4 and IL-13, were significantly decreased in patients compared with control subjects. In contrast, the pre-treatment levels of Th17-related cytokines, i.e., IL-17 and IL-23, were significantly increased in MABC patients. Interestingly, significantly higher levels of IFN-γ-induced protein (IP)-10 and monokine induced by IFN-γprotein (MIG) were detected in patients with failure of sputum conversion at post-treatment compared to patients with successful sputum conversion.

**Conclusion:**

Reduced Th1 and Th2 responses and enhanced Th17 responses in patients may perpetuate MABC lung disease, and the immunomolecules IP-10 and MIG, induced through IFN-γ, may serve as key markers for indicating the treatment outcome.

## Introduction

The prevalence of lung diseases caused by nontuberculous mycobacteria (NTM) is increasing worldwide, and NTM infections have been associated with environmental exposure to factors such as soil and water [1], [2]. *Mycobacterium abscessus* complex (MABC) represents a group of rapidly growing mycobacteria and an emerging cause of NTM lung disease in patients with cystic fibrosis and chronic lung diseases [3]–[5]. MABC comprises 3 subspecies; *M. abscessus* subsp. *abscessus* (hereafter referred to as *M. abscessus*), *M. abscessus* subsp. *massiliense* (hereafter *M. massiliense*), and *M. abscessus* subsp. *bolletii* [6]. Within MABC, *M. abscessus* is the most common pathogen, followed by *M. massiliense*, although the proportion of each subspecies varies according to geographical location [7], [8].

The outcome of natural infections with pathogenic mycobacteria ranges from early asymptomatic clearance to chronic clinical disease. MABC is the most drug-resistant of the mycobacterial pathogens, resulting in limited therapeutic options and a high treatment failure rate [8]. Although defects in immunological defense mechanisms have been suggested as predisposing factors to disease in a murine model of *M. abscessus* infection, including genetic defects in the interferon (IFN)-γ-interleukin (IL)-12 axis in MABC lung disease [9], understanding the nature of the immune response in humans to MABC at the molecular level is more important for the development of effective strategies to treat these mycobacterial species.

Although T helper type 1 (Th1) immunity typically plays a major role in controlling mycobacterial infections, host defense mechanisms against MABC remain poorly understood. *M. abscessus* induces the secretion of tumor necrosis factor (TNF)-α, IL-6, and IL-12p40 in murine macrophages via Toll-like receptor (TLR)-2 [10]. In addition, *M. massiliense* induces TNF-α and IL-6 production in murine macrophages [11]. Peripheral blood mononuclear cells (PBMCs) from NTM patients were shown to produce less Th1 cytokines (IFN-γ, IL-12, and TNF-α) compared to healthy controls, suggesting that NTM lung disease might reflect defects in the IL-12/IFN-γ pathway [12], [13]. Th2 immunity has been associated with poorer prognoses in TB but has not been described in NTM lung disease [14]. In addition, IL-17 levels are lower in patients with *M. avium* complex (MAC) lung disease compared to control subjects, suggesting that reduced Th17 immunity might be associated with MAC lung disease [15]. Thus, different host factors, such as cytokines and chemokines, have been implicated in determining the outcome of these infections and related treatment responses.

Recently, we reported that a lower level of Th1 and Th17 responses was associated with disease susceptibility in patients with MAC lung disease [16]. However, it remains unclear whether altered levels of these cytokines represent a general phenomenon in NTM infections or mycobacteria species-specific patterns. In the present study, we investigated the changes in host immune responses, including Th1, Th2, and Th17 immunity, after treating MABC lung disease patients with antibiotics.

## Methods

### Ethics statement

The data in the present study are part of an ongoing prospective observational cohort study investigating NTM lung disease (ClinicalTrials.gov Identifier: NCT00970801). The study protocol was approved through the institutional review board of the Samsung Medical Center (IRB approval 2008-09-016), and written informed consent was obtained from all participants.

### Study subjects

A total of 24 patients diagnosed with the nodular bronchiectatic or fibrocavitary forms of MABC lung disease at the Samsung Medical Center (Seoul, South Korea) were enrolled. A diagnosis of NTM lung disease was obtained for patients who fulfilled the clinical, radiographic, and microbiological diagnostic criteria of the American Thoracic Society [2]. The median age of the patients was 57.3 years (interquartile range [IQR] 50.3–64.8 years). Among the 24 patients identified as having MABC infection, 13 (54.2%) patients were identified as having *M. abscessus* infection, and the remaining 11 patients were identified as having *M. massiliense* infection. None of the patients had immunodeficiencies, malignancies, or were positive for antibodies to human immunodeficiency virus. The baseline characteristics of the patients are summarized in Table 1. Twenty-five healthy control subjects (10 men and 15 women) were recruited. None of the control subjects showed any evidence of pulmonary disease at the time of participation, and these individuals were non-smokers and free of allergic diseases, diabetes mellitus, liver diseases, and viral infections.

**Table 1:** Baseline characteristics of the 24 study patients with MABC lung disease.

	No. of patients (%) or median (IQR)
Age, years	57.5 (50.3–64.8)
Sex, female	16 (66.7)
Body mass index (kg/m^2^)	20.6 (18.8–21.8)
Smoking	
Non-smoker	19 (79.2)
Current or ex-smoker	5 (20.8)
Comorbidity	
Chronic heart disease	2 (8.3)
DM	2 (8.3)
Chronic liver disease	3 (12.5)
Rheumatic disease	1 (4.2)
Previous history of TB treatment	16 (66.7)
Etiology	
* M. abscessus*	13 (54.2)
* M. massiliense*	11 (45.8)
Type of diseases	
Nodular bronchiectatic form	19 (79.2)
Fibrocavitary form	5 (20.8)
Sputum culture conversion after treatment	15 (62.5)
Laboratory test at treatment	
CRP (mg/dL)	0.22 (0.05–1.04)
ESR (mm/hr)	42 (22–64)

MABC: *M. abscessus* complex; IQR: interquartile ranges; DM: diabetes mellitus; TB: tuberculosis; CRP: C-reactive protein; ESR: erythrocyte sedimentation rate.

Categorical variables were denoted as the No. of patients (%).

Continuous variables were denoted as median values (IQR).

### Serum samples

Serum samples were collected from healthy individuals at a single time point and from all patients before treatment and 12 months (IQR 11–12 months) after the start of antibiotic treatment. The samples were stored at −80°C until further testing.

### Multiplex bead-based cytokine assay

The serum samples were simultaneously screened for 22 cytokines using the Cytokine Assay kit (Panomics, Inc., Fremont, CA, USA) according to the manufacturer’s instructions. The plate was analyzed using a Luminex 100 instrument (Bio-Rad, Hercules, CA, USA). Each unique target molecule was assessed in duplicate.

### Statistical analysis

The data are presented as the median and IQR for continuous variables and as numbers (percentages) for categorical variables. The data were subjected to repeated measures analysis using PROC MIXED in SAS statistical software, version 9.1 (SAS Institute Inc., Cary, NC, USA). To compare patients and control subjects, Wilcoxon’s two-sample test or an independent two-sample t-test was used. To compare pre- and post-treatment conditions, a paired t-test or Wilcoxon’s signed rank test was used. To compare time points and changes between pre- and post-treatment conditions among subgroups, Wilcoxon’s two-sample test or an independent two-sample t-test was used. The *P*-value for differences in cytokine concentrations was corrected using Bonferroni's method to avoid inflated type I errors among 3 variables (pre-treatment, post-treatment, and changes between pre- and post-treatment conditions). A *P*-value of<0.05 was considered statistically significant.

## Results

### Th1 immunity-related immunomolecules in MABC lung disease

**Figure 1:**
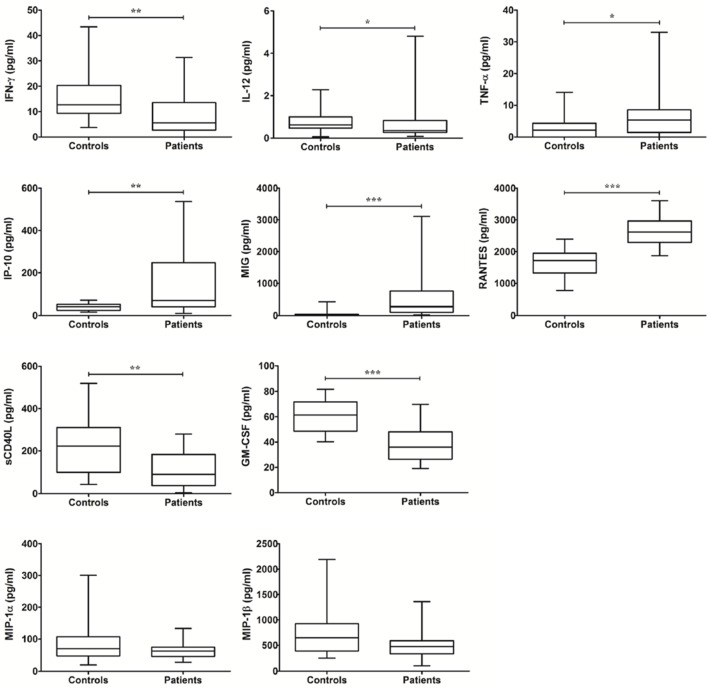
The levels of Th1 immunity-related cytokines between controls and patients at pre-treatment. The data are presented as box-and-whisker plots with the median, interquartile range, and minimum to maximum values. In comparison of patients and controls, Wilcoxon’s two sample test or independent two sample t-test is used.

To investigate host susceptibility factors, serum immunomolecules were first compared between healthy controls and patients with MABC lung disease prior to antibiotic treatment (see Table S1). The pre-treatment levels of IFN-γ and IL-12 were significantly lower in patients with MABC lung disease than control subjects, whereas the pre-treatment levels of TNF-α were upregulated in patients (Fig. 1). The levels of monokine induced by IFN-γ protein (MIG)/CXCL9, IFN-γ-induced protein (IP)-10/CXCL10, and regulated upon-activation normal T cell-expressed and secreted (RANTES)/CCL5 in patients with MABC lung disease were significantly higher than in control subjects, while the levels of sCD40L and granulocyte/macrophage colony stimulating factor (GM-CSF) were lower in patients than control subjects. The pre-treatment levels of macrophage inflammatory protein (MIP)-1α and MIP-1β in patients were not significantly different from those in control subjects (Fig. 1).

### Th2 immunity-related immunomolecules in MABC lung disease

**Figure 2:**
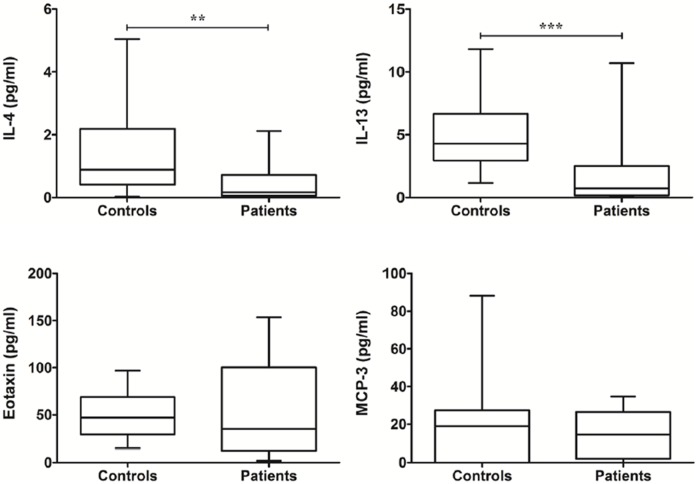
The levels of Th2 immunity-related cytokines between controls and patients at pre-treatment. The data are presented as box-and-whisker plots with the median, interquartile range, and minimum to maximum values. In comparison of patients and controls, Wilcoxon’s two sample test or independent two sample t-test is used.

The pre-treatment levels of IL-4 and IL-13, secreted from Th2 cells, were significantly lower in patients with MABC lung disease than in control subjects (Fig. 2). The pre-treatment levels of Eotaxin and monocyte chemotactic protein (MCP)-3 were not significantly different from those in control subjects (Fig. 2).

### Th17 immunity-related immunomolecules in MABC lung disease

**Figure 3:**
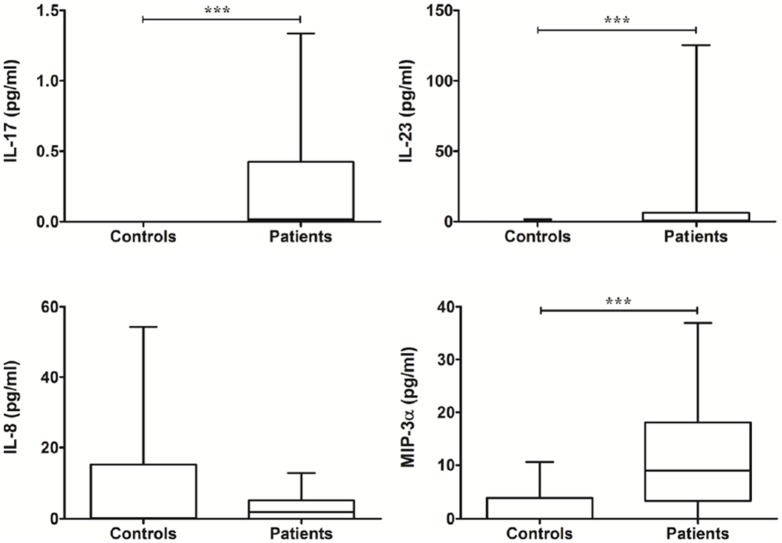
The levels of Th17 immunity-related cytokines between controls and patients at pre-treatment. The data are presented as box-and-whisker plots with the median, interquartile range, and minimum to maximum values. In comparison of patients and controls, Wilcoxon’s two sample test or independent two sample t-test is used.

The pre-treatment levels of IL-17 and IL-23 in patients with MABC lung disease were significantly higher than in control subjects (Fig. 3). The levels of MIP-3α/CCL20 in patients with MABC lung disease were significantly higher than those in control subjects, but no significant difference in the levels of IL-8 were observed between the two groups (Fig. 3).

### Changes in the cytokine levels between pre- and post-treatment conditions

All patients received combination antibiotic treatment for at least 12 months. We analyzed the changes in the cytokine levels during treatment, and Table 2 shows significant changes in the cytokine levels according to treatment.

**Table 2:** Overall comparison of serum immunomolecule levels in patients with MABC lung disease during antibiotic therapy.

Immunomolecules (pg/ml)	Patients		*p* Value^a^
	Pre-treatment	Post-treatment	
Th1-related			
IL-12	0.35 (0.27–0.83)	0.27 (0.17–0.45)	0.009
IFN-γ	5.60 (2.73–13.54)	2.99 (1.37–6.05)	0.001
TNF-α	5.30 (1.46–8.56)	3.24 (0.29–5.62)	0.046
MIP-1α/CCL3	62.24 (45.07–74.38)	45.41 (36.71–64.62)	0.004
MIP-1β/CCL4	471.5 (33.05–587.7)	404.1 (266.1–560.1)	0.020
RANTES/CCL5	2615 (2289–2615)	2455 (2104–2706)	NS
MIG/CXCL9	274.6 (106.0–756.4)	63.46 (31.31–348.9)	0.004
IP-10/CXCL10	70.76 (41.36–247.6)	34.37 (20.30–114.3)	0.006
sCD40L	88.38 (37.82–183.0)	22.77 (0.00–80.62)	0.009
GM-CSF	35.95 (26.43–48.02)	32.13 (24.05–44.34)	NS
Th2-related			
IL-4	0.16 (0.05–0.72)	0.16 (0.03–0.55)	NS
IL-13	0.72 (0.17–2.51)	1.30 (0.24–2.56)	NS
MCP-3/CCL7	14.51 (1.97–26.47)	13.73 (7.76–28.22)	NS
Eotaxin/CCL11	34.82 (12.32–100.2)	57.83 (22.20–85.46)	NS
Th17-related			
IL-17	0.02 (0.00–0.43)	0.00 (0.00–0.14)	0.039
IL-23	0.67 (0.00–6.36)	0.00 (0.00–9.13)	NS
MIP3α/CCL20	8.98 (3.24–18.10)	7.49 (3.25–15.30)	NS
IL-8/CXCL8	1.90 (0.00–5.05)	0.00 (0.00–2.35)	0.030
Others			
IL-10	0.48 (0.00–1.08)	0.00 (0.00–0.63)	NS
IL-27	817.4 (503.0–1051)	508.1 (344.6–783.9)	0.006
Adiponectin (ng/ml)	3425 (2455–5842)	4984 (2624–6944)	0.019
Leptin	2065 (413.3–3218)	2109 (512.6–4118)	NS

The data are presented as median values (IQR).

a Significance of the differences between pre- and post-treatment in patients. To compare pre- and post-treatment conditions, the paired t-test or Wilcoxon’s signed rank test was used. NS, not significant.

The levels of Th1-related cytokines and chemokines were significantly decreased after therapy. However, the levels of Th2-related molecules (IL-4, IL-13, Eotaxin, and MCP-1) were not changed after therapy. Th17-related molecules were either significantly decreased (IL-8 and IL-17) or remained unchanged (IL-23 and MIP-3α) according to treatment. Notably, adiponectin levels were increased only after treatment.

### Comparison of serum immunomolecule levels in patient subgroups

**Figure 4:**
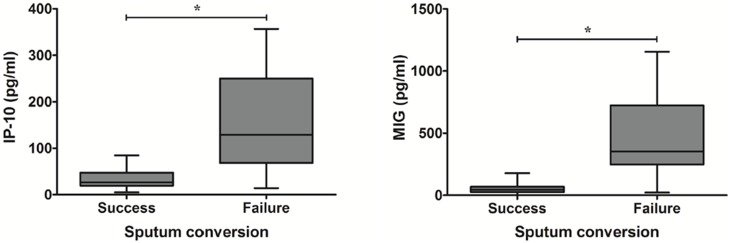
The post-treatment levels of IP-10 and MIG in patient subgroups according to sputum culture conversion [successes (*n* = 15) *versus* failures (*n* = 9)]. The data are presented as box-and-whisker plots with the median, interquartile range, and minimum to maximum values. To compare two subgroups, Wilcoxon’s two-sample test was used. *, *P*<0.05 for the results of the comparisons in patient subgroups according to sputum culture conversion.

Sputum culture conversion was achieved in 15 patients (62.5%). *M. abscessus* (*n* = 6) was the causative species in patients whose AFB cultures were not negatively converted. We also analyzed the changes in cytokine levels between pre- and post-treatment according to disease type (nodular bronchiectatic or fibrocavitary forms), negative conversion of sputum culture, and causative species (*M. abscessus* or *M. massiliense*). The cytokine levels between pre- and post-treatment were not different according to disease type and causative species (data not shown). However, the levels of IP-10 and MIG were lower in patients with successful sputum conversion than in patients with failed sputum conversion at post-treatment (Fig. 4).

## Discussion

NTM is becoming more prevalent as a cause of pulmonary disease in hosts without overt immunodeficiency. Although it remains unclear why otherwise healthy subjects should develop pulmonary diseases in response to these opportunistic pathogens, altered immunological factors have been associated with the development of disease. Therefore, we evaluated the levels of 22 immunomolecules that play critical roles in mycobacterial infection and observed significant differences in immunological responses between control subjects and patients during therapy.

Th1 immunity is important for protection against mycobacterial infections. The levels of Th1 immunity-related molecules, IFN-γ, IL-12, and sCD40L, were lower in patients at diagnosis time points. These findings support the idea that decreased IFN-γ, IL-12, and sCD40L levels are associated with MAC and *M. abscessus* lung disease [13], [16]. In contrast, the relative level of IL-10 was increased in the peripheral blood of patients compared with controls (*P* = 0.013). IL-10 is a regulatory cytokine involved in limiting the inflammatory response during NTM infection; in particular, IL-10 inhibits the secretion of IFN-γ and IL-12 in patients with MAC lung disease [17]. IL-27 is also associated with the Th1 response and is a critical factor in the induction of IL-10. The relative level of IL-27 was decreased in the peripheral blood of patients after treatment. Thus, the roles for IL-10 and IL-27 in the balance between pathology and protection are important. TNF-α is also an essential cytokine for the development of protective immunity. In the present study, the production of TNF-α was significantly increased in the peripheral blood of patients and decreased during therapy. Indeed, the levels of TNF-α have been shown to be higher in patients with tuberculosis (TB) [18]. Notably, TNF-α is produced in both Th1 and Th17 cells in response to *M. abscessus* infection, and the pattern of TNF-α was similar to that of IL-17, which is produced by Th17 cells. In the present study, IL-17 production was significantly increased in patients with MABC lung disease and decreased during therapy. Therefore, increased TNF-α in patients with MABC lung disease is more likely produced by Th17 cells than Th1 cells. In addition, a high level of TNF-α in MABC patients might reflect a compensatory host defense mechanism to control mycobacterial infection. CXCR3 (receptor for IP-10 and MIG) and CCR5 (receptor for MIP-1α, MIP-1β, and RANTES) are predominantly expressed by Th1 cells [19]. The IP-10, MIG, and RANTES levels were higher in the peripheral blood of patients than in control subjects. Increased levels of IP-10 and MIG in *M. tuberculosis*-infected human cells and an increased level of RANTES in bronchoalveolar lavage fluid from TB patients have previously been reported [20], [21]. Thus, these inflammatory chemokines may be increased in response to MABC infection.

Th2 cells express CCR3 (receptor for Eotaxin and MCP-3), and Eotaxin therefore plays a key role in Th2 cell recruitment. The levels of Th2-related cytokines and chemokines were decreased in the peripheral blood of patients and were not changed during therapy. In MAC patients, the levels of IL-4 and IL-13 were not significantly different from those in control subjects and were not changed during therapy [16]. Thus, defective Th2 immunity might be associated with host susceptibility to MABC infection.

We also examined Th17 immunity in the present study. To our knowledge, this study is the first to evaluate changes in cytokine levels associated with Th17 immunity in response to MABC infection in humans. Th17-related cytokines have been informative for studying protective and damaging immune responses. For instance, it has been reported that IL-23 restores immunity to *M. tuberculosis* infection and preferentially promotes the production of IL-17 [22]. The potent pro-inflammatory cytokine IL-17 induces the expression of chemokines that promote neutrophil recruitment and granuloma organization throughout infection. A recent study showed increased IL-17 and IL-23 gene expression in the lungs of patients with active TB [23] and the study by Lim et al. reported that MAC lung disease was associated with defects or biases in Th1 and Th17 immunity [15]. An increase of Th2 and Th17 cells was observed in peripheral blood of patients with chronic obstructive pulmonary disease in comparison to controls, while Th1 cells was not significantly different [24]. In addition, increased Th17 response was observed in peripheral blood of patients with cystic fibrosis, pulmonary sarcoidosis and allergic asthma [25]–[27]. Thus, the elevated Th17 response in combination with Th1 or Th2 immunity plays an important role in disease development and progression of non-infection-mediated lung disease as well as mycobacterial lung disease.

In the present study, the pre-treatment levels of IL-17 and IL-23 were higher in the peripheral blood of patients than in control subjects, and IL-17 production was decreased after treatment. Thus, the increased production of these cytokines in response to MABC infection might not be sufficient to suppress the development of disease. IL-23 was also shown to increase IFN-γ and IL-17 expression in the lungs of IL-12(p40)-deficient mice. Therefore, the induction of IL-17 by Th17 cells might be inadequate for protection, and increased IL-17 levels in MABC patients might instead play a pathological role.

Leptin-deficient mice show reduced lung IFN-γ levels and greater susceptibility to *M. tuberculosis* compared with wild-type mice [28], and the levels of leptin were shown to be lower in TB patients than control subjects [29]. In the present study, the leptin levels in the peripheral blood were not different between patients with MABC lung disease and control subjects, and no change was detected during therapy (Table 2). In TB patients, the adiponectin levels did not change after treatment [30], whereas our results showed that the adiponectin levels in the peripheral blood of patients were increased after treatment.

In addition, the levels of each cytokine between pre- and post-treatment were not different in all subgroups according to patterns of disease and causative species. However, the levels of IP-10 and MIG were significantly lower in the peripheral blood of patients with successful sputum conversion than in patients with failed sputum conversion at post-treatment. Furthermore, the levels of these chemokines in patients with successful sputum conversion were similar to those observed at baseline after treatment. In previous studies, IP-10 and MIG levels were significantly higher in TB patients compared to control subjects, suggesting that these chemokines are potential biomarkers of TB infection [31]–[33]. In particular, IP-10 is expressed at very high levels along with less biodegradable potency in a whole blood sample compared with IFN-γ, the current diagnostic marker in TB patients [34]–[36]. Thus, IP-10 may serve as a biomarker for mycobacterial lung disease.

IFN-γ induces IP-10 and MIG production; however, other cytokines, such as TNF-α and IL-17, have been implicated in the induction of IP-10 and MIG production [37], [38]. In the present study, the pattern of IP-10 and MIG production was similar to that of TNF-α and IL-17, and the levels of these cytokines were significantly increased in the peripheral blood of patients and decreased during therapy. This finding might explain why MABC patients show high levels of IP-10 and MIG but lower levels of IFN-γ. Indeed, elevated plasma IP-10 levels have been associated with poor responses to treatment in TB and NTM patients [32], [34], [39], [40]. Thus, measuring the IP-10 and MIG levels might facilitate the assessment of treatment efficacy.

The present study has some limitations. First, this study was conducted at a single center and performed on a referral basis, with the analysis of only a small number of Korean patients. Second, this study was preliminary because we did not investigate the precise mechanism of the specific molecules associated with clinical outcomes. Finally, it is unable to be determined in our study whether our results were the cause of the development of MABC infection or whether MAC infection caused the alterations of the cytokine levels. Alterations in serum immunomolecule levels may be the reflection of MABC infection. The further studies are required to evaluate whether the altered level of cytokines are the cause or result of MABC infection. Nevertheless, the cytokines detected in the study during antibiotic therapy may help in the rational design of more effective therapeutic strategies against MABC lung disease.

In conclusion, reduced Th1 and Th2 responses and enhanced Th17 responses in patients might perpetuate MABC lung disease. The balance of the type of T cell immunity is critical for determining susceptibility to MABC and the outcome of disease. Improved knowledge of the function of each cytokine during the initiation of immune responses and after treatment will facilitate the development of better immunological and therapeutic interventions to promote balanced immune responses and predict treatment outcomes.

## Supporting Information

Table S1 **Overall comparison of serum immunomolecule levels between controls and patients with MABC lung disease at pre-treatment.**
(DOC)
