# Supplementary material for: Importance of Reciprocal Balance of T Cell Immunity in Mycobacterium abscessus Complex Lung Disease
Source: PLoS One. 2014 Oct 8;9(10):e109941. doi: 10.1371/journal.pone.0109941 (PMC4190320; doi:10.1371/journal.pone.0109941)
Supplement: Table S1 — Overall comparison of serum immunomolecule levels between controls and patients with MABC lung disease at pre-treatment. (DOC) [file pone.0109941.s001.doc]

**Supplementary Table 1.** Overall comparison of serum immunomolecule levels between controls and patients with MABC lung disease at pre-treatment.

| Immunomolecules (pg/ml) | Controls | Patients | *P*-value^a^ |
| --- | --- | --- | --- |
| Th1-related  IL-12 | 0.62 (0.47–1.00) | 0.35 (0.27–0.83) | 0.039 |
| IFN-γ | 12.68 (9.30–20.37) | 5.60 (2.73–13.54) | 0.003 |
| TNF-α | 2.18 (0.00–4.32) | 5.30 (1.46–8.56) | 0.022 |
| MIP-1α/CCL3 | 69.19 (46.48–107.1) | 62.24 (45.07–74.38) | 0.248 |
| MIP-1β/CCL4 | 646.8 (388.0-924.5) | 471.5 (33.05–587.7) | 0.063 |
| RANTES/CCL5 | 1716 (1323–1947) | 2615 (2289-2615) | <0.001 |
| MIG/CXCL9 | 11.84 (2.67–35.78) | 274.6 (106.0-756.4) | <0.001 |
| IP-10/CXCL10 | 41.42 (24.57–52.94) | 70.76 (41.36-247.6) | 0.004 |
| sCD40L | 221.9 (98.27–310.5) | 88.38 (37.82– 183.0) | 0.004 |
|  |  |  |  |
| Th2-related  IL-4 | 0.89 (0.41–2.19) | 0.16 (0.05–0.72) | 0.001 |
| IL-13 | 4.28 (2.96–6.66) | 0.72 (0.17–2.51) | <0.001 |
| GM-CSF | 61.41 (48.60–71.66) | 35.95 (26.43–48.02) | <0.001 |
| MCP-3/CCL7 | 18.91 (0.00-27.35) | 14.51 (1.97-26.47) | 0.779 |
| Eotaxin/CCL11 | 46.85 (29.19-68.73) | 34.82 (12.32-100.2) | 0.599 |
|  |  |  |  |
| Th17-related  IL-17 | 0.00 (0.00–0.00) | 0.02 (0.00–0.43) | <0.001 |
| IL-23 | 0.00 (0.00–0.00) | 0.67 (0.00–6.36) | <0.001 |
| MIP3α/CCL20 | 0.00 (0.00–3.81) | 8.98 (3.24-18.10) | <0.001 |
| IL-8/CXCL8 | 0.16 (0.00–15.21) | 1.90 (0.00-5.05) | 0.483 |
|  |  |  |  |
| Others  IL-10 | 0.00 (0.00–0.00) | 0.48 (0.00–1.08) | 0.013 |
| IL-27 | 465.7 (297.2-1389) | 817.4 (503.0-1051) | 0.159 |
| Adiponectin (ng/ml) | 3273 (1992-5942) | 3425 (2455-5842) | 0.612 |
| Leptin | 2181 (1747-2889) | 2065 (413.3-3218) | 0.399 |

The data are presented as median (IQR).

^a^ Significance of differences between healthy controls and patients before treatment. In comparison of patients and controls, Wilcoxon’s two sample test or independent two sample t-test is used.
